# Mesenchymal stem cells as professional actors in gastrointestinal cancer therapy: From Naïve to genetically modified

**DOI:** 10.22038/ijbms.2021.54735.12277

**Published:** 2021-05

**Authors:** Mehrdad Nasrollahzadeh Sabet, Masood Movahedi asl, Mahtab Kazemi Esfeh, Navid Nasrabadi, Maryam Shakarami, Behrang Alani, Asma Alimolaie, Sara Azhdari, Ebrahim Cheraghi

**Affiliations:** 1 School of Medicine, AJA University of Medical Science, Tehran, Iran; 2 Non-Communicable Diseases Research Center, Endocrinology and Metabolism Research Institute, Tehran University of Medical Sciences, Tehran, Iran; 3 Department of Biology, School of Science, Shiraz University, Shiraz, Iran; 4 School of Medicine, Shahid Beheshti University of Medical Sciences, Tehran, Iran; 5 Department of Cellular & Molecular Biology, Isfahan University, Isfahan, Iran; 6 Department of Applied Cell Sciences, Faculty of Medicine, Kashan University of Medical Sciences, Kashan, Iran; 7 Department of Biology, Faculty of Science, Shahid Bahonar University of Kerman, Kerman, Iran; 8 Department of Anatomy and Embryology, School of Medicine, Bam University of Medical Sciences, Bam, Iran; 9 Department of Biology, Faculty of Sciences, University of Qom, Qom, Iran

**Keywords:** Cancer therapy, Gastrointestinal cancers, Gene therapy, Genetic engineering, Mesenchymal stem cells

## Abstract

Considering the high incidence and mortality rate of gastrointestinal cancers (GIs) worldwide and partial success of the current available GI cancer treatments, there is a necessity to discover more effective approaches in cancer therapy. The failure in conventional therapies seems to be related to the resistance of cancer cells to chemotherapy, inability to target tumor cells especially in metastatic cancers, deficient drug concentrations in tumor sites, and unfavorable effects on pivotal non-malignant bodily tissues following systemic administration. In this context, we need an appropriate carrier for the delivery of therapeutic agents specifically to the GI cancer site. Mesenchymal stem cells (MSCs), a prominent cell-based strategy for cancer treatment, overcome various cancer therapy limitations and could be used as vehicles to deliver many anticancer agents such as therapeutic genes (DNA or interference RNA), oncolytic viruses, and chemotherapeutic or nanoparticle drugs. Moreover, secreted molecules of unmodified MSCs lead to deregulation of several proteins and different signaling pathways eradicating cancer cells. In the present review, at first, we overview the characteristics and utility of MSCs in cancer therapy, secondly, we discuss the application of naïve MSCs and utilization of MSCs in the delivery of therapeutic agents in GI cancer therapy and, finally, more information about harnessing of genetically modified MSCs in GI cancer treatment will be presented.

## Introduction

According the World Health Organization (WHO) statistics, cancer was the second leading cause of death globally and it is estimated that 18.1 million worldwide new cases of cancer were responsible for 9.6 million deaths in 2018 (1, 2). Gastrointestinal (GI) cancers include a gamut of 36 malignancies with five major types of esophagus, stomach, liver, pancreas, and colorectal carcinoma. It has been estimated that these types of cancers occurred in 4.8 million new cases in 2018 worldwide of which 3.4 million died. These figures accounted for 26% and 35% of all global cancer incidence and cancer-related deaths, in 2018, respectively (3). To date, there are several conventional types of cancer therapy especially for GI cancers, including surgical resections, radiation therapy, chemotherapy, and hormonal therapy which usually require to be used in combination; although, they have remained unsuccessful in many instances (4). The failure in these conventional therapies seems to be related to the resistance of cancer cells to chemotherapy, inability to target tumor cells especially in metastatic cancers, deficient drug concentrations in tumor sites, and unfavorable effects on pivotal non-malignant bodily tissues following systemic administration (5). These limitations justified the necessity to discover more effective approaches in anti-tumor therapy*. *With the recent signs of progress towards understanding the molecular basis of carcinogenesis, targeted therapies have been established and are currently being employed in clinics as anti-cancer therapy (6, 7). In this context, drugs can destroy cancer cells without affecting normal cells by targeting and/or interfering with specific molecules that are involved in the initiation and progression of cancer cells (7). Cancer gene therapy as one of the approaches in the field of targeted therapy includes different methods such as gene transfer and oncolytic virotherapy (8). In gene transfer strategy, new genes are introduced into a cancerous cell or the surrounding tissue to cause cell death or stimulate the immune system to destroy cancer cells (9). Some of these genes comprise anti-angiogenic, apoptosis inducer, cytokines, chemokines, and suicide genes (10). An oncolytic virus can selectively replicate and cause cell destruction without harming normal tissues (11). Delivering to the specified target requires the vehicles that encapsulate the genes, drugs, or oncolytic viruses and place them particularly on the cancer cell and cancer local environment of the tumor. Cellular vectors have numerous advantages subsuming low immunogenicity, relative safety for clinical gene therapy (low risk of mutagenesis), lack of DNA insert size limitation, low cost, and ease of manufacturing (12, 13). Cell-based vectors are vehicles with the capability of delivering many anticancer agents encompassing small molecule drugs, DNA or interference RNA, proteins, suicide genes, nanoparticles, and oncolytic viruses (14). Mesenchymal stem cells (MSCs) could be utilized as the vehicle in cancer therapy; MSCs have low immunogenic potential on the account of not stimulating the immune system (15). MSCs have tumor tropism; that is, they migrate to sites of injury and inflammation, transferring the genes and their products just into the milieu of the tumor. This process reduces the side effects of systemic toxicity and could achieve more efficient gene delivery to the target (16). On the other hand, some studies demonstrated that naïve MSCs could be used in different diseases especially in cancer treatment (17). In the present review, we addressed four topics: first, an overview of characteristics and benefits of MSCs in cancer therapy; second, application of naïve MSCs in GI cancer therapy; third, utilization of these cells in drug and oncolytic virus delivery in GI cancer therapy, and finally, harnessing of genetically modified MSCs in GI cancer eradication.

***The aptitude of MSCs in cancer therapy***

Adult stem (AS) cells divide into neural stem cells (NSCs) (ectoderm), hematopoietic stem cells (HSCs) (mesoderm), and MSCs (mesoderm) and have a great potential in regenerative medicine (18). MSCs can be isolated from various human tissues, including bone marrow, umbilical cord blood, placenta, adipose tissue, skin, tooth pulp, synovium, and peripheral blood (19). These cells can retain their characteristics such as proliferative ability and differentiation efficiency during higher passages. Also, MSCs have the capacity to differentiate into all three lineages namely ectoderm (e.g., neural), mesoderm (e.g., osteoblasts, chondrocytes, and adipocytes), and endoderm (e.g., hepatic and pancreatic) (20). Some characteristics such as immunosuppressive and anti-inflammatory properties have made MSCs a promising cell source for tissue engineering; these cells express a low level of surface human leukocyte antigen (HLA) class I but do not express HLA class II (which can activate alloreactive T cells), co-stimulatory molecules CD80, CD86, CD134, CD252, CD40, and CD40 ligand (21, 22). These features reduce the risk of graft versus host rejection due to avoidance of the immune response. Also, among minimal criteria for recognition of MSCs isolated from all sources, we refer to the plastic-adherence ability of these cells when grown *in vitro* and stable expression of surface antigens CD73, CD90, and CD105 (21, 23). Studies have demonstrated that MSCs can migrate towards injured, inflamed, or cancerous tissues (24). Further experiments revealed that several factors are involved in the mediation of MSC tropism to a specific malignant site. They showed that in the tumor microenvironment (TME) there is a chemotactic gradient consisting of different chemokines, cytokines, and growth factors including CCL2, CCL5, CXCL12, CXCL13, CXCL16, NT-3, GM-CSF, G-CSF, VEGF, MCP1, MIP-1α, TGF-β, SCF-c-Kit, HGF/c- Met, SDF-1, TNF-α, IL-8, IL-1β, IL-6, IL-3, and UPAR, which are able to recruit MSCs. Also, expression profiling of MSCs revealed the existence of receptors such as CCR1, CCR2, CCR3, CCR4, CCR6, CCR7, CCR8, CCR9, CCR10, CXCR1, CXCR2, CXCR3, CXCR4, CXCR5, CXCR6, RAGE, CX3CR1, VEGFR, and c-met (25, 26). Among these molecules, SDF-1/CXCR4 and CXCL12/CXCR4 axes accomplish the central role in the migration of MSCs (27, 28). Furthermore, adhesion molecules and matrix metalloproteinases such as VLA-4, VCAM-1, ICAM-1, P and L-selectin, b1- and b2-integrins, MMP-1, MMP-2, and MMP-9 are implicated in MSCs migration and homing (25, 29). As previously stated, MSCs, either manipulated or naïve (unmodified), have beneficial characteristics consisting of natural tumor-trophic migration property, easy isolation from different sources, self-renewal capacity, multi-lineage differentiation capacity, non host immune response stimulant and consequently useful in allogeneic injection, efficient transduction with viral or non-viral vectors containing a target gene, and ability to secrete of therapeutic molecules directly or in exosomes. All of these characteristics make these cells suitable cell therapy vehicles for the delivery of agents into tumor cells (30). 

What’s more, several studies reported antitumor properties of naïve MSCs in cancer treatment in both *in vitro* and different animal models of malignancies which are attributed to the factors released by these cells. For instance, some experiments demonstrated that naïve MSCs inhibit glioma, melanoma, leukemias, hepatocellular carcinoma (HCC), colorectal, breast, and other GI cancers that will be discussed in the next section (31-33). Naïve MSCs with their secreted molecules lead to deregulation of several proteins and different signaling pathways such as down-regulation of FAK, X-linked inhibitor of apoptosis protein (XIAP), Akt, PI3K, NFkB, and Wnt pathway, or up-regulation of PTEN and TRAIL which ultimately results in tumor eradication (34, 35). However, in different studies, MSCs have been considered as cells with double-bladed effects in the suppression or progression of tumors (36). Therefore, the usage of genetically engineered MSCs as a vehicle to deliver biological agents at the tumor site could lead to improved anti-cancer effect of these cells.

***Naïve MSCs in GI cancer therapy***

Several studies on different types of GI cancers represented that unmodified MSCs have the ability to prevent excessive cell growth when co-cultured with tumor cells *in vitro* and *in vivo*. In this section, we will overview the application of these cells in the eradication of GI cancer cells (Table 1).

***Colorectal cancer (CRC)***

For analysis of the effect of human MSC-derived bioactive molecules on CRC cell proliferation, Paiboon *et al*. used the conditioned media derived from different hMSC sources to culture the HT29 cell line. Their results indicated that this media condition, regardless of the source of MSCs, can meaningfully decrease cell proliferation. Their further analysis established that only the ≤50 kDa fraction (50, 30, 10, and <10 kDa fractions) of MSCs-conditioned media could reduce the proliferation of HT29 cells. Besides, Paiboon *et al*. reported that bone marrow-derived MSCs (BMSCs) have superior migratory ability to HT29 cells rather than other sources such as chorion-derived MSCs (ChMSCs), amniotic-derived MSCs (AMSCs), or umbilical cord-derived MSCs (UCMSCs) (37).

Several lines of evidence unveiled that MSC-derived exosomes can deliver therapeutic proteins, mRNA, and microRNAs to tumor cells and have therapeutic potential for cancer. A study revealed the lower and higher expression miR-4461 and its target gene (COPB2), respectively, in different CRC cell lines (HCT116 and SW480) than that of normal cells. Further analysis uncovered that BM-MSCs-derived exosomal miR-4461 might hamper cell proliferation, migration, and invasion by down-regulating the expression of the COPB2 gene (38). One study on methylnitronitrosoguanidine (MNNG)-induced CRC rat model and cell line unraveled that BMSCs could decrease CRC initiation by immune system modulation rather than through a direct effect on the cancer cells. In detail, MSCs strikingly diminished both tumor initiation and progression as well as increased lifespan in the rat model. Moreover, MSCs administration not only limited impairment of healthy tissues but also reduced tumor growth following radiotherapy *in vivo*. In addition, MSCs in cell lines had a slightly protective effect on MNNG-mediated oxidative stress, DNA damage, and also cell proliferation after severe MNNG exposure (39). Two different studies on the colitis-associated colorectal cancer (CAC) model demonstrated that MSCs with an effect on the immune system can lead to tumor inhibition. They induced the development of colitis-associated CRC in mice model by azoxymethane and dextran sulfate sodium. Researchers injected BMSCs into these mice models and observed reduced tumor cells which were derived by alleviated expression of proinﬂammatory cytokines such as TNFa, IL-6, and IL-1b and reduction of STAT3 activation by phosphorylation in colorectal tissues (40). Similarly, research revealed that intravenous injection of UCMSCs decreases inflammatory cytokines in colon tissues and serum and also prompts the differentiation of Treg cells through the Smad2 signaling pathway and consequently represses colitis and inhibits the development of CAC *in vivo* (41). 

***Hepatocellular cancer (HCC)***

According to the previously mentioned study performed by Paiboon *et al.*, there were similar results about the effect of MSCs on C3A cells (HCC cell line), but the effect of fractionated MSCs secretome on C3A was different from HT29. They disclosed that only the 100 kDa fraction of various sources of MSCs conditioned media can reduce the proliferation of HCC cells (37). By co-culturing of BMSCs with HepG2, researchers revealed that BMSCs can inhibit and induce proliferation and apoptosis of cancer cells, by secretion of Dkk-1 from BMSCs and, therefore, inhibit expression of bcl-2, c-Myc, survivin, and β-catenin in the HCC cell line. In this study, also, the anti-tumor effect of BMSCs was confirmed in nude mice after co-injection of BMSCs and HepG2 (42). Similar results were obtained in research by Qiao *et al*. who worked on a co-culture system of H7402 (HCC cell line), HepG2 cells, and gestational tissue-derived MSCs as well as SCID mice model transplanted with MSCs and H7402 cells (43).

Two studies with co-culturing of adipose tissue-derived MSCs (ATMSCs) and different HCC cell lines reported inhibition of cell proliferation, migration, and invasion and induction of cell apoptosis. It seems that these therapeutic effects emanate from inhibition of c-Myc, hTERT, and Akt signaling and induction of P53, retinoblastoma (Rb), and tissue inhibitor metalloproteinases TIMP-1, TIMP-2, and TIMP-3 (44, 45). A study discovered that co-culturing of ATMSCs with HCC cells or treatment of these cells with ATMSCs conditioned medium (ATMSCs-CM) leads to inhibition of cell growth in correlation with augmented protein levels of p53/p21, phosphorylation of STAT1, and decreased proliferating cell nuclear antigen (PCNA) level. Furthermore, they demonstrated that treatment of these co-cultures with anti-IFN-β antibody and JAK1/JAK2 inhibitors leads to reversal of the inhibitory effect of ATMSCs. This leads the authors to conclude that these therapeutic effects were through the IFN-β→JAK/STAT1 pathway (46). Ma and coworkers evaluated the anti-tumor activity of BMSCs pulsed with homologous tumor-derived exosomes (TEX) and IFN-γ and unveiled that this strategy can augment the antitumor activity of MSCs (47). Research revealed that co-culturing of BMSCs with MHCC97-H cells (HCC cell line) and *in vivo* analysis have contradictory results. They reported that *in vitro* MSCs inhibit invasiveness but enhance cell proliferation, and in *in vivo* assay mice have a larger tumors but cellular numbers of lung metastases were reduced (48). 

One experiment evaluated the antitumor activity of ATMSCs exosomes on a rat model of HCC and discovered that these exosomes can suppress HCC via promoting natural killer T-cell (NKT-cell) antitumor responses (49). Another study showed that ATMSCs can increase the efﬁcacy of radiotherapy in the eradication of HCC cells at *in vitro* and *in vivo* levels (50). Also, according to two similar studies, a combination of chemotherapy (with Sorafenib) and placenta-derived MSCs (PMSCs) might lead to inhibition of cell proliferation, tumor growth, and angiogenesis and induction of tumor necrosis and apoptosis in *in vitro* and *in vivo* milieu (51, 52). 

***Pancreatic cancer***

Considering the importance and therapeutic effects of MSCs-derived exosomes, a study demonstrated that exosomes derived from human UCMSCs can suppress cell proliferation, invasion, and increase apoptosis of pancreatic ductal adenocarcinoma (PDAC) by delivering exogenous miR-145. Moreover, they represented that intra-tumoral injection of UCMSC exosomes in subcutaneous xenograft tumor increases tumor eradication (53). Two groups of researchers by working on human ATMSCs and rat UCMSCs, respectively, independently uncovered that these cells, after co-culture with pancreatic cancer cell lines, inhibit cell proliferation, colony size, and number and induce of apoptosis and G0/G1 arrest. Also, *in vivo* analysis revealed that injection of these cells into a mouse model leads to inhibition of tumor growth and higher survival time (54, 55).

***Other GI cancers***

To the best of our knowledge, there was just one article that evaluated the therapeutic effect of MSCs on cholangiocarcinoma (bile duct cancer). They demonstrated that hUCMSCs can inhibit the growth of cholangiocarcinoma xenograft tumors. In addition, *in vitro* analysis revealed that conditioned media from hUCMSCs may inhibit proliferation and induce apoptosis of HCCC-9810 (cholangiocarcinoma cell line). Further analysis unveiled that this inhibitory effect was on the account of the decrement in the phosphorylation and activation of PDK1 and Akt, which in turn leads to a reduction in the phosphorylation of GSK3b. As a consequence, activation of GSK3b resulted in lower free cellular β-catenin levels, decreased β-catenin translocation to the nucleus, and, ultimately, increased apoptosis (56).

Researchers reported that co-culturing of UCMSCs with EC1 cells (esophageal cancer cell line) might suppress cancer cell proliferation and induce apoptosis; consistently, they discovered that this strategy reduces stemness capacity of cancer cells due to down-regulated expression of stem cell markers such as KLF4, OCT2, SOX2, CXCR4, and CXCR7 (57). Intriguingly, a study evaluated the effect of artificial cell fusion of MSCs with esophageal carcinoma cells on tumorigenesis. On the one hand, they reported increased apoptosis and benign trans-differentiation compared with reprogramming to cancer stem cells; whilst on the other hand, they showed that augmented expression of DUSP6/MKP3 in the MAPK pathway was responsible for induction of apoptosis and suppression of growth suppression, both *in vitro* and *in vivo* (58). A study on oral cancer manifested that a combination of Cisplatin + BMSCs in the nude mice model of oral squamous cell carcinoma (OSCC) leads to small tumor size, minimal hypoxia status, and induced apoptosis (59). According to one research, the therapeutic effect of gingival-derived MSCs (GMSCs) as another type of MSCs was mediated by expression regulation of some pro-apoptotic and anti-apoptotic genes such as up-regulation of p-JNK, Bax, cleaved PARP, and cleaved caspase-3, and down-regulation of PCNA, CDK4, Bcl-2, cyclin D1, p-ERK1/2, and survivin. These results were visible in direct co-culture of GMSCs with oral cancer cells and also in indirect co-culture systems with conditioned medium derived from GMSCs; besides, the anticancer effect of these cells was observed when co-injected with oral cancer cells to BALB/c nude mice (60). Intra-tumoral injection of exosomes derived from UCMSCs resulted in the inhibition of angiogenesis and tumor growth of OSCC in Syrian golden hamsters (61).

***MSCs for drug delivery in GI cancer therapy***

Low targeting efficiency and systemic toxicity limit the applications of anticancer agents such as nanoparticles and chemotherapy. Nowadays, by considering the innate ability of MSCs to deliver a variety of therapeutic interventions, drug delivery has been accompanied by some improvements, especially in GI cancer therapy. 

A study introduced MSCs as promising targeted nanocarriers for CRC treatment. In this study, they reported that when doxorubicin (DOX)-loaded superparamagnetic iron oxide (SPIO) nanoparticles (NPs) are coated with MSCs membranes, it increases the antitumor effect and decreases the unwanted immune system response compared with DOX-SPIO-only and control groups (62). A large body of evidence indicated both high resistance of MSCs to anti-neoplastic drugs including paclitaxel (PTX), doxorubicin (DXR), and gemcitabine (GCB) and the ability of MSCs in the delivery of antineoplastic drugs to cancer cells. It has been documented that OSCC and MSCs from gingival papilla (GinPa-MSCs) cells can properly be loaded with PTX, DXR, and GCB, and consequently secrete an active form of drugs in satisfactory volume, which results in significant malignant cell eradication (63). In another study on GinPa-MSCs, it was revealed that these cells can uptake and liberate PTX and have an anti-cancer activity effect on pancreatic cancer cells *in vitro* (64). In two similar studies published on GinPa-MSCs and BMSCs, researchers reported that these cells could uptake PTX and then release the active form of this drug by extracellular vesicles (EVs), suggesting that PTX with incorporation in exosome biogenesis are involved in pancreatic cancer therapy (65, 66). Same results were extracted from another study where they immortalized ATMSCs with hTERT+SV40 (TS) genes and confirmed the role of microvesicles (MVs) in the delivery of cytotoxic chemotherapeutic drugs (67). Besides, researchers in two different studies with work on human AMSCs and BMMSCs loaded with PTX and GCB demonstrated the anti-proliferation ability of this strategy on pancreatic cancer *in vitro* and *in vivo* (68, 69).

***MSCs for oncolytic virus delivery in GI cancer therapy***

Oncolytic virotherapy, a new cancer therapy strategy, is a potentially hopeful alternative to conventional cancer treatment; however, as mentioned in the first section, there are hitherto some limitations. These barriers include viral tropism, antiviral immunity, systemic toxicity, and delivery strategy. MSCs with their intrinsic ability in tumor homing provide competent vehicles for oncolytic viruses to diminish any risk of systemic administration (i.e., systemic toxicity) of the naked virus. 

A group working on hepatocellular carcinoma (HCC) unraveled that human BMSCs could be transduced by HCC-oncolytic adenovirus (HCC-oAd) and this virus can replicate effectively in these cells. *In vivo* analysis revealed that HCC-oAd/MSC might lyse HCC cells (Hep3B). To create an orthotopic HCC tumor model, Hep3B cells were injected into the liver of athymic nude mice. Then, after 35 days, systemic delivery of HCC-oAd /MSC gave rise to significantly reduced tumor growth without obvious toxicity on normal organs (70). Conditionally replicative adenovirus (CRAd) as a new approach in oncolytic virotherapy has the speciﬁcally self-amplification capacity in tumor cells under the control of a tumor speciﬁc promoter without triggering the immune reactions. Researchers generated a CRAd that harbored an E1A gene under the control of the α-fetoprotein promoter and microRNA-122 target sequence. After the transduction of CRAd into human UCMSCs and tumor homing, these cells differentiated into hepatocyte-like cells inside TME, and subsequently CRAds were replicated, packaged, and released strikingly in the tumor site which eventually led to a lysate of cancerous liver cells in orthotopic liver tumor xenograft mouse model (71). Several studies reported that the measles virus (MV) has potent selective oncolytic activity through inducing extensive cytopathic effects (CPE) against human cancers especially in GI cancer. Although MV can just replicate in tumor cells, insufficient delivery of this virus to cancer cells is known as the major hurdle (72, 73). A study carried out transduction of MV to human BMSCs and also injected HCC cells orthotopically into SCID mice. Their results displayed that BMSCs by heterofusion can transfer MV infectivity to HCC *in vitro* . Inline, *in vivo* analysis showed that these cells can obviously inhibit tumor growth not only in measles antibody-naïve but also in passively-immunized SCID mice; while the antitumor activity of naked MV viruses was just observed in measles antibody-naïve SCID mice (74). The application of human BMSCs in the delivery of oAds and their effect on the inhibition of tumor growth was evaluated. OAd-infected BMSCs could migrate to PDA spheroids *in vitro* and significantly decrease tumor volume, Ki67 (proliferation marker), and CD24 (progression marker) expression and increase necrotic morphology in cancer cells (75).

***Genetically engineered MSCs***

**Figure 1:**
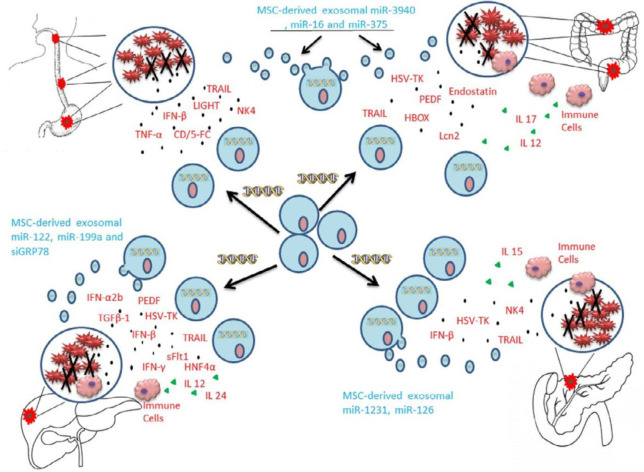
Application of genetically engineered MSCs for delivery of therapeutic factors in different types of GI cancers

Recently, several experiments recruited MSCs as a delivery vehicle in cancer gene therapy because of their characteristics, including comparative ease of isolation and expansion, relative ease of genetic modifications, low immunogenicity, tumor tropism, and the ability for the secretion of therapeutic molecules directly or with exosomes in the tumor site. These cells can transfer multiple genes with different functions such as antiangiogenic, pro-apoptotic, cytokines and chemokines (for cancer immunotherapy), suicide genes, miRNAs, and siRNAs (16, 76). As depicted in Figure 1, genetically engineered MSCs are used for the delivery of several therapeutic factors in different types of GI cancers. In this section, we will overview these studies and their achievements in GI cancer therapy.

***Colorectal cancer***

The first study that surveyed the application of genetically modified MSCs in CRC therapy was performed by Luetzkendorf *et al*. in 2009; they assessed the usage of BMSCs in the delivery of TNF-related apoptosis-inducing ligand (TRAIL) as a proapoptotic factor in CRC therapy (77). Several studies have worked on the application of TRAIL in cancer therapy; this gene belongs to the TNF family and is capable of inducing apoptosis just in tumorigenic or transformed cells (78). Multiple researchers reported the anticancer potential of human TRAIL; but, *in vivo* application of this gene was limited because of short half-life in the circulatory system owing to quick clearance by the kidneys and, therefore, reaching of insufficient amount of this protein to the tumor site (79, 80). Luetzkendorf *et al*. accomplished the transduction of BMSCs with a lentiviral vector containing the TRAIL gene. These cells stably expressed highly TRAIL protein without any change in MSC characteristics. The co-culture of these cells with traditional CRC cell lines increased apoptosis in cancer cells; furthermore, the same results were obtained from co-culturing with CRC-cell lines that are resistant to TRAIL (soluble form) such as HCT-8 and SW480. *In vivo* analysis indicated that subcutaneous delivery of a high proportion of BMSCs/ TRAIL increases apoptosis and consequently reduces tumor growth (77). 

In the next study, Grisendi *et al*. transduced ATMSCs with full-length-encoding retroviral vectors and observed higher cell apoptosis in different cell lines with increased caspase-8 activation. According to *in vivo* analysis, after subcutaneous and intravenous injection of ATMSCs/TRAIL, these cells, without obvious side effects on normal organs, migrated to the tumor site, significantly induced apoptosis, and decreased tumor size (80). In 2013, it was reported that, in contrast with chemotherapy only, a combination of human BMSCs/TRAIL and low doses of chemotherapy with 5-ﬂuorouracil (5-FU) increased, meaningfully, the apoptosis of CRC cells (HCT116). Also, they reached the same results in HCT116 xenografts with intravenous injection of BMSCs/TRAIL and intraperitoneal injection of 5-FU. Furthermore, their analysis delineated that after induction of apoptosis by secretable TRAIL (sTRAIL), expression of TRAIL-receptor 2 (TRAIL-R2) was increased in cancer cells. The next analysis implemented on the knocking-down of TRAIL-R1 and TRAIL-R2 clones revealed a significantly higher level of apoptosis in TRAIL-R1 knockdown cells, leading the authors to conclude that TRAIL-R2 was responsible for TRAIL-induced apoptosis. These results instigated them to generate two types of MSCs which transduced with two types of sTRAIL, including sTRAIL^DR5^ (selective for TRAIL-R2) and sTRAIL^DR4^ (selective for TRAIL-R1). They found that BMSCs/sTRAIL^DR5^ lead to significantly induced apoptosis in combination with 5-FU treatment *in vivo*. Additionally, the application of BMSCs/TRAIL on HCT116 p53 (-/-) cells disclosed that apoptosis induction is p53-independent (81). YUAN *et al*. compared the cancer apoptosis efficacy of full-length human TRAIL (ﬂf) and soluble form of TRAIL (sT) that were delivered by BMSCs. Their findings unraveled that BMSC/ﬂT have higher efficacy in destruction of cancer cells compared with BMSC/sT because BMSC/ﬂT expressed both cell-surface and soluble form of TRAIL, as well as these transduced cells, could induce apoptosis in malignant cells which are resistant to recombinant TRAIL (82).

The existing evidence clearly suggests that high-mobility group box 1 (HMGB1) has different potentials in cancer progressions such as mounting the growth, angiogenesis, migration, invasion, metastasis, and inhibition of apoptosis by affecting different signaling pathways (83, 84). Further to this, evidence documented that inhibition of HMGB1 leads to suppression of tumor growth (85). The binding of ABOX as an antagonist of HMGB1 to the receptor of HMGB1 named receptor for advanced glycation end products (RAGE) leads to inhibition of HMGB1 properties in cancer progression, especially in angiogenesis (86). Kikuchi *et al*. generated BMSCs/ABOX and conducted the co-culture of these cells with endothelial cells which were stimulated via supernatant from SW480 cell culture; they observed a significant decline in endothelial cell migration compared with control. Moreover, systemic administration of MSCs resulted in noticeable lower tumor size in the human colon cancer-bearing mouse xenograft model because of the anti-angiogenic ability of secreted ABOX from engineered MSCs (87). 

The role of lipocalin 2 (Lcn2) in cancer is controversial in this sense some studies referred to its function in cancer promotion and others emphasized the anticancer ability of this molecule especially in suppression of invasion, angiogenesis, and metastasis in different types of cancers. According to one publication, after the transduction of this gene in BMSCs, Lcn2 was overexpressed and intravenous injection of BMSCs/Lcn2 in the mouse model of colorectal cancer led to a reduction in liver metastasis and expression of VEGF in liver tissue (88). Pigment epithelium-derived factor (PEDF) is another anticancer protein that inhibits tumor development especially with an anti-angiogenic property which leads toward suppression of tumor growth and metastasis in several malignancies (89, 90). PEDF usually is expressed in normal tissues and acts as an antagonist of VEGF-A (91). A study unraveled that genetically modified BMSCs which overexpress PEDF (BMSCs/PEDF) for a prolonged time can meaningfully suppress tumor metastasis and malignant ascites formation in a mouse model of CRC through reduction of angiogenesis and increased cell apoptosis (92). Two different works were conducted on the application of MSC-derived exosomes in the delivery of miRNAs and the antitumor effect in CRC. The initial findings of Xu *et al*., using dual-luciferase reporter gene assay, indicated that miR-16 was significantly down-regulated in CRC cells and targeted the ITGA2 gene. Afterward, they transfected BMSCs with miR-16 and co-cultured CRC cells with miR-16-overexpressing exosomes which were secreted by engineered BMSCs. These exosomes could suppress proliferation, migration, and invasion, and increase cell apoptosis. Likewise, *in vivo* analysis revealed that these exosomes could reduce tumor growth in the nude mice model (93). A similar study discovered that co-culture of CRC cells with MSC-derived exosomes which overexpress miR-3940 reduces cell invasion and epithelial-mesenchymal transition (EMT) *in vitro*; consistently, *in vivo* analysis showed reduced tumor volume and metastasis. Concerning the role of overexpressed ITGA6 in CRC progression, further evaluation indicated that the antitumor effect of miR-3940-carrying exosomes was through targeting of this gene (94). Several studies unveiled that sodium iodide symporter (NIS) stimulates the transportation of iodide from the extracellular space into the thyroid cell and is applicable in the diagnostic and therapeutic use of radioiodine. Transduction of MSCs with NIS has antitumor ability when using ^131^I or 188Re and can be monitored by whole-body imaging using ^124^I PET. Application of ^131^I in a mice model which was bearing transduced MSCs with NIS under the control of RANTES/CCL5-promoter led to decreased tumor growth and increased overall survival (95). Researchers uncovered applications of HSV-TK as a suicide gene in cancer gene therapy. The HSV-TK gene codes a thymidine kinase that is able to convert nucleoside analogs such as ganciclovir (GCV) into cytotoxic molecules. Researchers transduced MSCs with HSV1-TK under the control of the tetracycline-inducible system (Tet-system) which expressed the suicide gene in the presence of doxycycline (DOX). They reported the antitumor ability of HSV1-TK with the Tet-On system for colon cancer both *in vitro* and *in vivo* (96). Accumulating evidence emphasizes the antiangiogenic capability of endostatin via suppression of Wnt signaling pathways in different cancer types (97, 98). The expression of endostatin in PMSCs, which was transduced using adenovirus, resulted in the reduction of angiogenic ability in CRC cell lines, decreased the number of tumor nodules, and increased tumor cell apoptosis and survival time in the mice model (99).

Several pieces of evidence have indicated that direct administration of immunostimulatory cytokines for cancer treatment has some limitations. Hence, precisely targeted delivery of cytokines via genetically modified MSCs would be a better alternative with decreased adverse drawbacks. Researchers carried out the first study in genetic modification of MSCs to overexpress IL7 and IL12; they demonstrated that these cytokines could enhance the efficacy of CAR T cells in CRC treatment through promoting homeostatic expansion and Th1 polarization (100). In Table 2, we summarized studies that worked on the application of genetically modified MSCs in CRC therapy. 

***Hepatocellular cancer***

Several works have accomplished the application of genetically modified MSCs in the treatment of HCC; these studies demonstrated antitumor effects, including inhibition of cell proliferation and tumor growth, cell invasion, angiogenesis, metastasis, and boosting of apoptosis in HCC cell lines *in vivo*. In this section and in Table 3, we will review the application and therapeutic effect of this strategy in HCC treatment. 

Researchers transduced IL12 in BMSCs with adenoviral vectors and injected these cells into the mice model of HCC; subsequently, they observed prevention in the tumor formation in about 92% of samples. However, the rates of tumor formation and progression in mice models with the injection of naked adenovirus encoding IL-12 (AdIL-12) and naïve MSCs were about 83% and 100%, respectively (101). Exploring the application of human UCMSCs in delivery of IL24 for HCC therapy, researchers infected UCMSCs with Ad-IL24 under the control of human telomerase reverse transcriptase (hTERT) promoter which is overexpressed in cancer cells but not in somatic cells and subsequently reported inhibition of cell growth in cell line and xenograft HCC model. Besides, they observed that this inhibitory effect was enhanced by low doses of 5-Fu (102). Researchers generated genetically modified BMSCs that co-expressed IL-10 and IFN-γ and their *in vitro* analysis signified that co-culturing of these BMSCs with HepG2 cells leads to decreased cell viability via increased expression of some tumor suppressors such as p21 and p27 and suppressed expression of the CCND1 gene. Meanwhile, the subcutaneous delivery in the HCC xenograft model caused a reduction in the tumor volume (103). In another effort for immunotherapy of HCC with MSCs, IFN-b was transduced in BMSCs and these cells were co-cultured with HepG2 and Huh7 cells. The results revealed the decrease and increase, respectively, in the cell proliferation and cell cycle which is mediated via inhibition of AKT/FOXO3a. Likewise, *in vivo* analysis represented the suppression and enforcing of tumor growth and survival, respectively, in the NOD/SCID mice tumor model (104). Compelling evidence has signified that Interferon-α (IFN-α) could be used as a therapeutic cytokine in HCC therapy (105, 106). For reducing the side effect and increasing the half-life and concentration of IFN-α2b, a study employed BMSCs to deliver this cytokine in the HCC tumor site. Stable expression of IFN-α2b in the IFN-α2b gene-modiﬁed BMSCs resulted in inhibition of HCC cell proliferation and increased cell cycle arrest in HCC cell lines (HepG2 and Huh7) and also suppression of tumor growth *in vivo* via repression of the Notch signaling pathway (107). Transforming growth factor-beta (TGFβ) is a multi-functional cytokine with anticancer effects (108, 109). A study revealed that co-culturing of BMSCs-TGFβ-1 with hepatoma cells results in increased cell proliferation; although, the migration ability of these cells was inhibited. Furthermore, the administration of BMSCs-TGFβ-1 cells in the MHCC97-H and MHCC97-L xenograft mouse model inhibited tumor growth (110). Some studies have addressed the application of modified MSCs-derived exosomes in HCC therapy. The resistance of HCC tumors to chemotherapeutic agents such as sorafenib and doxorubicin has been well-established. Overwhelming evidence has pointed out that up-regulated Grp78 is involved in the resistance of multiple cancer types to sorafenib (111, 112). A novel study reported that BMSCs could be genetically modified to secrete exosomal siGRP78 and discovered that these exosomes combined with sorafenib can down-regulate GRP78 expression and decrease HCC cell proliferation and invasion *in vitro* and also suppress tumor growth and metastasis in the mice model (113). Lou *et al*. transduced miR-122 and miR-199a in ATMSCs in two different experiments and observed that ATMSC-exosomes-199a can profoundly sensitize HCC cells (PLC/PRF/5 cell line) to doxorubicin via inhibition of mTOR pathway and consequently increase tumor cell killing. These results also were confirmed in the BALB/c nude mice model (114). Another study reported that ATMSC-exosomes-miR-122 might increase the sensitivity of HCC cells to sorafenib *in vitro* and *in vivo* (115). 

Through inhibiting VEGF, researchers created a modified murine BMSCs to overexpress sFlt1 (soluble Fms-like tyrosine kinase-1) as an angiogenesis inhibitor. They elucidated that the combination of this engendered MSCs with low-dose doxorubicin deterred HepG2 cell proliferation and tumor growth in the HepG2 xenograft mouse model (116). Other research groups delineated that intravenous injection of BMSC-PEDF in the HCC orthotopic nude mouse model decreases tumor volume, angiogenesis, and lung metastasis (117). 

Transduction of NIS in MSCs under the control of a hypoxia-responsive promoter, when using ^124^I and ^123^I, could be useful in tomography for HCC. Also, systematic injection of a therapeutic dose of ^131^I + MSCs –NIS inhibited tumor growth and increased survival of the orthotopic HCC mouse model (118). According to a study, the secretion of hepatocyte nuclear factor 4a (HNF4a) from MSCs which was transduced with lentiviral vectors in culture media, and delivery of conditioned medium from these cells could decrease HCC cell proliferation and metastasis *in vitro* and could inhibit tumor growth in nude mice. Expression analysis manifested that this growth inhibitory effect was related to the suppression of the Wnt/b-catenin signaling pathway (119). Researchers created HSV-Tk-infected MSCs with two different promoters named Tie2 and CCL5. By analogy with Tie2/HSV-TK-MSCs, after injection of these cells in the HCC mice model, CCL5/HSV-TK-MSC had more achievements in HCC tumor eradication, revealing that application of different promoter sequence is important in the anti-cancer effect of this strategy (120). 

To induce apoptosis in HCC cells, some studies generated TRAIL-MSCs and evaluated the cancer-killing ability of this strategy *in vitro* and *in vivo*. For instance, researchers inserted secretable trimeric TRAIL (stTRAIL) into BMSCs and co-cultured these cells with heat-shock-treated liver cancer cells and illustrated significantly increased cancer cell apoptosis through induction of caspase-3 expression. Moreover, decreased tumor growth and increased survival rate in the nude mice model were seen in *in vivo* analysis (121). A similar study, with leveraging stTRAIL-MSCs and TRAIL-MSCs (a non-secreting form of TRAIL+ MSCs), uncovered HCC cell apoptosis in co-culturing (direct culture) with modified MSCs and with conditioned media (indirect culture) of modified MSCs. They found that amount of cell apoptosis has a direct relationship with MSC/HepG2 ratio (122). Convergent lines of evidence have delineated that apoptin could kill cancer cells with little toxicity outcome on normal cells. Apoptin is a chicken anemia virus-derived protein and can induce apoptosis in cancer cells in a selective manner (123, 124). An *in vitro* analysis indicated that genetically engineered MSCs with apoptin could meaningfully suppress cancer cell proliferation in direct and indirect co-culture (125).

***Pancreatic cancer***

Harnessing of MSCs in gene therapy of pancreatic cancer has been investigated by inserting several genes such as IL15, IFN- β, miRNAs (miR-126 and miR-1231), TRAIL, HSV-TK, and NK4 into MSCs to release encoded products directly or indirectly with exosomes. In Table 4, we summarized studies that utilized genetically modified MSCs in pancreatic cancer gene therapy.

For the first time, Grisendi and coworkers evaluated the application of MSCs in the delivery of TRAIL to induce apoptosis of pancreatic cancer cells. They transduced full-length human TRAIL with retroviral vectors into the isolated human ATMSCs and, in combination with bortezomib as a chemotherapeutic drug that induces caspase-8, observed an antitumor effect *in vitro*. Furthermore, they obtained similar results when intravenously or subcutaneously injecting these genetically engineered MSCs into the mouse model of pancreatic cancer (80). In the next study, researchers created engineered MSCs to overexpress and secrete sTRAIL and disclosed that this therapy approach in combination with inhibition of XIAP (an anti-apoptotic protein) by RNA interference (RNAi) leads to significant inhibition of metastatic growth cell line *in vivo* (126). Similarly, modified sTRAIL^DR5^- released MSCs, in combination with silencing of XIAP, could kill PancTu1, a TRAIL-resistant pancreatic cancer cell line (81). Han and colleagues indicated that the transfection efficiency of sTRAIL into human MSCs could be increased by utilization of photochemical internalization (PCI) and creation of polyplexes using sTRAIL plasmid and branched polyethyleneimine (bPEI). In the same vein, they displayed a higher expression of sTRAIL and eradication of tumors in the xenograft mouse model (127). Antitumor activity of modified MSCs which overexpress and release sTRAIL was assessed by measuring the induction of apoptosis in pancreatic ductal adenocarcinoma (PDAC) cell lines and reduction of tumor size in tumor-bearing NOD/SCID mice (128). Having considered the fact that low expression of some miRNAs in pancreatic cancer is associated with tumor progression, some researchers decided to exploit these molecules in pancreatic cancer therapy. Researchers transfected BMSCs with miR-1231 oligonucleotides and then co-cultured pancreatic cancer cells with exosomes derived from supernatants of these modified stem cells; their findings showed inhibition in the proliferation, migration, and invasion of cancer cells. Besides, intravenous injection of these therapeutic exosomes to the BALB/C nude mice model resulted in a decline in tumor growth (129). In another study, *in vitro* and *in vivo* analysis demonstrated that restoration of miR-126 expression in pancreatic cancer cells via genetically engineered BMSCs-exosomes leads to suppression of cancer cell proliferation, invasion, and metastasis as well as induction of apoptosis by inhibition of ADAM9, a gene that is necessary for cancer progression (130, 131). Two distinct experiments used C57BL/6 mice BMSCs for suicide gene therapy by the mediation of HSV-TK. One of them used CCL5 promoter and reported a 50% decrease of primary pancreatic tumor growth and also alleviation of liver metastases to about 60% (132). In the other study, researchers via designing HSV-TK under the control of Tie2 promoter enhancer in BMSCs and mice model of pancreatic cancer elucidated decreased tumor growth and increased survival (133).

Another study reported that engendered human IFN-β-producing BMSCs can migrate to the tumor site and suppress tumor growth in the orthotopic human pancreatic cancer mouse model after intraperitoneal injection. Additionally, they demonstrated that treatment with an anti-inﬂammatory agent such as CDDO-Me leads to lessening of the tumor-homing ability of MSCs and as a consequence their antitumor effects (134). A study revealed that systemic administration of MSCs, which is isolated from human umbilical cord blood and release IL15 (UCMSCs- IL15), to the mouse model of pancreatic cancer might lead to a reduction in the tumor growth and induce survival time through NK and CD8 T cell activation and proliferation (135). Transduction of rat BMSCs with adenovirus containing the NK4 sequence, an antagonist of hepatocyte growth factor (HGF) and strong inhibitor of angiogenesis, and co-culture of these modified cells with SW1990 (a pancreatic cancer cell line) resulted in significant inhibition of proliferation and migration (136).

***Other GI cancers***

Some studies have scrutinized the application of modified MSCs in other GI cancer (oral cancer, esophageal cancer, and gastric cancer) therapies through insertion of different genes in MSCs isolated from different sources such as bone marrow, gingival, and umbilical cord. In Table 5 we alluded to the application and therapeutic effect of this strategy in other GI cancer treatments.

A group of researchers engineered MSCs to overexpress TRAIL under the control of the tetracycline promoter. According to *in vitro* analysis, these cells increased apoptosis in human tongue squamous cell carcinoma (TSCC) (H357) cells; relevantly, subcutaneous xenograft evaluation revealed that this approach drives strong tumor growth inhibition and significantly reduces lung metastases (137). The next study evaluated GMSCs that were transduced with TRAIL by a lentiviral vector; they reported enhanced cell death and apoptosis *in vitro* and decreased or, in some cases, inhibited TSCC growth *in vivo* (138). Expression and secretion of IFN-β by modified MSCs reduced and increased proliferation and apoptosis of CAL27 cells (TSCC cell line), respectively. Furthermore, these results were confirmed in the mouse model, manifested by decreased tumor volume and the number of cancer cells (139). 

In a study researchers transduced BMSCs with TRAIL- expressing adenoviral vector to examine the antitumor activity of these cells on esophageal cancer cells both *in vitro* and *in vivo*. These engineered stem cells inhibited proliferation and increased apoptosis of esophageal cancer cells (Eca-109 cells) and repressed tumor growth in the Eca-109 xenograft mouse model (140). With regard to down-regulation of miR-375 in esophageal squamous cell carcinoma, researchers transfected miR-375 in human UCMSCs and indicated that exosomes derived from these cells might inhibit esophageal cancer cell proliferation, invasion, migration, tumorsphere formation, and increase apoptosis in esophageal squamous cell carcinoma (ESCC) cell lines; also, exosomes blocked tumor growth in the subcutaneous xenograft tumor model (141). 

Co-culture of cytosine deaminase (CD)-expressing BMSCs with 5-fluorocytosine (5-FC) and systemic administration on gastric cancer cell uncovered conversion of 5-FC to 5-FU and anticancer ability of this approach. These results also were repeated in nude mice bearing MKN45 tumors after intravenous injection of CD-BMSC (142). In another study, full-length human TNF-α was transduced into MSCs; after subcutaneous injection of SGC-7901 (gastric cancer cells) and UCMSC-TNF-α in the mouse model, tumor growth was significantly decreased (143). Based on currently available evidence, LIGHT (TNFSF14; a member of the tumor necrosis factor (TNF) receptor superfamily) has a central role in regulation of antitumor immunity by co-stimulating the proliferation of T cells and activating apoptosis of different tumor cells (144, 145). In a study they created genetically modified UCMSCs to overexpress the LIGHT gene (UCMSC-LIGHT) and reported that these cells can strongly suppress tumor growth by increasing tumor necrosis and apoptosis (146). Other experiments revealed that BMSC-NK4 can migrate and inhibit gastric cancer in cell culture (MKN45) and systemic administration of these cells in gastric cancer xenografts causes strong tumor growth inhibition (147).

***Future perspective***

Currently, it is estimated that GI cancers have 26% and 35% of all global cancer incidence and cancer-related deaths, respectively (3). To date, there are several conventional types of cancer therapy, especially for GI cancers; but, because of several limitations such as inability to target tumors, deficient drug concentrations in tumor sites, and unfavorable effects on crucial non-malignant tissues, these approaches have led to failure and increased mortality due to these types of cancers. Therefore, there is a necessity to discover more effective approaches in anti-tumor therapy, especially for GI cancers. In this way, we need an appropriate carrier to specifically deliver the therapeutic gene to the cancer site. MSCs, naïve or engineered, have the capability to be utilized as a vehicle to deliver countless anticancer agents such as therapeutic genes, oncolytic viruses, and chemotherapeutic drugs for treatment of a variety of cancers, especially GI cancer. The possible positive role of MSCs in tumor progression should be taken into account. Altogether, more research is required for better comprehension of MSCs biology and application of these cells in GI cancer therapy.

**Table 1:** Application of unmodified MSCs in GI cancer therapy

**Cancer type**	**Cell line or mouse model**	**Source of MSC**	**Anti-cancer effects**	**Ref**
CRC	HT29 cells	Human UCMSCs, AMSCs, and ChMSCs	Suppressed cell proliferation	(37)
CRC	C57BL/6 female mice	Mice BMSCs	Inhibited tumorigenesis of inflammatory bowel disease	(40)
CRC	C57BL/6 male mice	Human UCMSCs	Induced differentiation of Treg cells and therefore inhibited colitis and suppressed the development of CAC.	(41)
CRC	CC531 cells and Sprague Dawley (SD) rats	Human BMSCs	Reduced both cancer initiation and cancer progression also extended lifespan.	(39)
CRC	HCT116 and SW480	Human BMSCs	BMSCs-derived exosome miR-4461 inhibited cell migration and invasion.	(38)
HCC	C3A cells	Human UCMSCs, AMSCs, and ChMSCs	suppressed cell proliferation	(37)
HCC	male Fischer-344 (F344) rats	Rat ATMSCs	ATMSC-derived exosomes suppressed HCC by promoting NKT-cell antitumor responses.	(49)
HCC	HepG2 cells and BALB/c female mice	Human BMSCs	Inhibited proliferation and increased apoptosis *in vitro* and inhibited tumor growth *in vivo*	(42)
HCC	H7402, HepG2 cells and SCID mice	Z3 human MSCs	Increased time for tumor formation and decreased tumor size.	(43)
HCC	MHCC97-H and nude mice	Human BMSCs	Inhibited invasiveness and numbers of lung metastases	(48)
HCC	HepG2, Huh7, SMMC7721, and Bel7402 cells	Human ATMSCs	Inhibited cell proliferation, and increased cell apoptosis	(44)
HCC	HepG2 and PLC-PRF-5 cells	Human ATMSCs	Inhibited cell proliferation, migration, invasion, and increased cell apoptosis	(45)
HCC	MHCC97-H cells and BALB/c nude mice	Human BMSCs	Reduced tumor tissue weight especially 3 weeks after BMSCs engraftment and decreased metastatic potential and increased apoptosis	(148)
HCC	HepG2 and HuH7cells and male BALB/c nude mice	Human ATMSCs	Increased inhibitory effect of radiotherapy and reduced cell growth, migration, and invasion both *in vivo* and *in vitro*.	(50)
HCC	Huh7 cells	Human ATMSCs	Inhibited cell growth	(46)
HCC	H22 cells	Mice BMSCs	Inhibited cell proliferation	(47)
HCC	HepG2 cells	Human UCMSCs	Inhibited cell growth and increased cell apoptosis	(149)
HCC	HepG2 cells and male athymic nude mice (nu/nu; C57BL/6)	Human PMSCs	Combination of chemotherapy (sorafenib) and MSC reduced cell proliferation, increased tumor necrosis and apoptotic-positive cells	(51)
HCC	HepG2 cells and male athymic nude mice (nu/nu; C57BL/6)	Human PMSCs	MSCs in combination with Sorafenib inhibited tumor growth, proliferation, angiogenesis, and increased apoptosis	(52)
CCCs	HCCC-9810 cells and BALB/c nude mice	Human UCMSCs	Inhibited tumor growth *in vivo* also inhibited proliferation and induced apoptosis of tumor cells *in vitro*	(56)
PC^4^	Capan-1, CFPAC-1, BxPC-3, and Panc-1 cells, and male BALB/c nude mice	Human UCMSCs	Inhibited PDAC cell proliferation and invasion, and increased apoptosis and cell cycle arrest, also reduced growth of xenograft tumors *in vivo.*	(53)
PC	Capan-1, Capan-2, BxPC-3, Miapaca-2 and Panc-1 cells and C57BL/6 nude	Human ATMSCs	Induced cancer cell necrosis and G1-phase arrest, and inhibited tumor growth in a mouse model	(54)
PC	PAN02 cells and wild-type female C57BL/6 mice	Rat UCMSCs	Increased G1-phase arrest, inhibited cell proliferation, colony size, and number, also decreased growth and enhanced survival time *in vivo*	(55)
ESCC	EC1 cells	Human UCMSCs	Inhibited proliferation, promoted apoptosis, and reduced the stemness capacity	(57)
ESCC	EC9706 cells and SCID mice	Human UCMSCs	Induced apoptosis *in vitro* and reduced tumor formation *in vivo*.	(58)
OC	C57BL/6 nude	Mice BMSCs	Reduced hypoxia status and increased apoptotic activity in tumor tissues	(59)
OC	CAL27, WSU-HN6 cells, and male BALB/c nude mice	Human GMSCs	Suppressed cell growth and induced apoptosis *in vitro* and *in vivo*	(60)
OC	Syrian golden hamsters	Human UCMSCs	Antitumor effect after intra-tumoral injection correlated with a loss of tumor vasculature.	(61)

CRC: Colorectal cancer; HCC: Hepatocellular carcinoma; CCCs: Cholangiocarcinomas; PC: Pancreatic cancer; ESCC: Esophageal squamous cell carcinoma; OC: Oral cancer; UCMSCs: Umbilical cord-derived MSCs; BMSCs: Bone marrow-derived MSCs; ATMSCs: Adipose tissue-derived MSCs; ChMSCs: Chorion-derived MSCs; PMSCs: Placenta-derived MSCs; AMSCs: Amniotic-derived MSCs; GMSCs: Gingival-derived MSCs; MSCs: Mesenchymal stem cells; GI cancer: Gastrointestinal cancers

MSCs: Mesenchymal stem cells; GI cancer: Gastrointestinal cancers

**Table 2:** Application of genetically modified MSCs in CRC therapy

**Transfected gene**	**Source of MSCs**	**Vector**	**Anti-cancer effects**	**ref**
ABOX	Human BMSCs	Plasmid	Suppressed angiogenesis and decreased tumor size	(87)
HSV-TK	Human BMSCs	Retrovirus	Increased cancer cell eradication *in vitro* and *in vivo*	(96)
Lipocalin 2 (Lcn2)	Human BMSCs	Plasmid	Inhibited liver metastasis of colon cancer in nude mice.	(88)
miR-16	Human BMSCs	Plasmid	Inhibited proliferation, migration, and invasion, and also increased apoptosis of the CRC cells.	(93)
miR-3940	Human UCMSCs	plasmid	Inhibited invasion and EMT of CRC cells as well as growth and metastasis of tumors *in vivo*.	(94)
RANTES-NIS	Human BMSCs	plasmid	RANTES-NIS-MSC + ^131^I decreased tumor growth and increased overall survival	(95)
TRAIL	Human ATMSCs	Retrovirus	Increased apoptosis *in vitro* and *in vivo*	(80)
TRAIL	Human BMSCs	Plasmid	TRAIL-MSC increased apoptosis in TRAIL sensitive and resistant cells	(150)
TRAIL	Human BMSCs	Adenovirus	BMSCs/sTRAIL^DR5^ through TRAIL-R2, combination with 5-FU result in p53-independent increased apoptotic effect, *in vitro* and *in vivo*.	(81)
TRAIL	Human BMSCs	Lentivirus	MSC-ﬂTRAIL cells demonstrated high cancer eradication efﬁciency compared with MSC-sTRAIL.	(82)
TRAIL	Human BMSCs	Lentivirus	TRAIL-MSC increased apoptosis in TRAIL sensitive and resistant cells also inhibited tumors *in vivo*.	(77)
Endostatin	Human PMSCs	Adenovirus	Reduced angiogenesis *in vitro* and decreased tumor nodules and tumor cell proliferation also increased cell apoptosis and survival *in vivo*.	(99)
IL7 and IL12	Human BMSCs	Retrovirus	Improved the efficacy of CAR T cells in the treatment of solid malignancies	(100)
PEDF	Mice BMSCs	Adenovirus	Inhibited tumor angiogenesis, inducing apoptosis.	(92)

BMSCs: Bone marrow-derived MSCs; UCMSCs: Umbilical cord-derived MSCs; ATMSCs: Adipose tissue-derived MSCs; PMSCs: Placenta-derived MSCs

MSCs: Mesenchymal stem cells; CRC: Colorectal cancer

**Table 3:** Application of genetically modified MSCs in HCC therapy

**Transfected gene**	**Source of MSCs**	**Vector**	**Anti-cancer effects**	**ref**
HSV-TK	Human BMSCs	Plasmid	Reduced tumor growth in mice model and CCL5/HSV-TK-MSCs was more applicable in HCC therapy	(120)
PEDF	Human BMSCs	Lentivirus	Inhibited tumor growth, microvessel density, and metastasis	(117)
sFIT-1	Mice BMSCs	Adenovirus	Combination of MSC- sFlt1 and low dose doxorubicin inhibited HCC *in vitro* and *in vivo*	(116)
HIF-NIS	Human BMSCs	Plasmid	systematic injection of a therapeutic dose of ^131^I + MSCs –NIS leads to inhibition of tumor growth and increased survival *in vivo*	(118)
IL12	Mice BMSCs	Adenovirus	Inhibited tumor formation in mice model	(101)
IFN-b	Human BMSCs	Plasmid	Lower growth rate *in vitro*. Inhibited HCC growth and increased the survival time *in vivo*.	(104)
siGRP78	Human BMSCs	Plasmid	Inhibited HCC combined with sorafenib *in vitro* and *in vivo*	(113)
HNF4α	Human UCMSCs	Lentivirus	Inhibited hepatoma cell proliferation and metastasis *in vitro*. Also inhibited tumor growth *in vivo*.	(119)
miR-122	Human ATMSCs	Plasmid	Enhanced chemosensitivity	(115)
TRAIL	Rat BMSCs	Lentivirus	Increased apoptosis in heat-shock-treated liver cancer cells *in vitro* and decreased tumor growth and increased survival time *in vivo*.	(121)
TGFβ-1	Human BMSCs	Lentivirus	Promoted hepatoma cell proliferation and inhibited hepatoma cell migration *in vitro* and *in vivo*.	(110)
IFN-γ and IL-10	Rat BMSCs	Lentivirus	inhibited HCC *in vitro* and *in vivo* by modulating cell cycle regulators and MAPK pathway	(103)
IFN-b	Human BMSCs	Retrovirus	Inhibited the proliferation of HCC cells *in vitro* and decreased tumor growth *in vivo*.	(104)
IFN-α2b	Human BMSCs	Plasmid	Inhibited HCC cell growth through negatively regulating the Notch signaling pathway	(107)
miR-199a	Human ATMSCs	Lentivirus	Enhanced chemosensitivity.	(114)
sFlt-1	Human BMSCs	Lentivirus	Reduced microvessel density in mice also inhibited tumor growth and prolonged survival in an HCC mouse model via systemic injection.	(151)
Apoptin	Human BMSCs	Adenovirus	Inhibited proliferation of liver cancer cells (HepG2).	(125)
IL24	Human UCMSCs	Adenovirus	Inhibited HepG2 cell growth, and this inhibitory effect was enhanced by low doses of 5-Fu.	(102)
TRAIL	Human Pancreas	Plasmid	Inhibited cell proliferation	(122)
TRAIL	Human BMSCs	Lentivirus	Inhibited HCC in combination with chemotherapeutic agents (cisplatin) *in vivo*.	(152)

BMSCs: Bone marrow-derived MSCs; UCMSCs: Umbilical cord-derived MSCs; ATMSCs: Adipose tissue-derived MSCs; MSCs: Mesenchymal stem cells; CRC: Colorectal cancer

**Table 4:** Application of genetically modified MSCs in pancreatic cancer therapy

**Transfected gene**	**Source of MSCs**	**Vector**	**Anti-cancer effects**	**ref**
TRAIL	Human BMSCs	Adenovirus	Genetically modified MSCs in combination with XIAP inhibition suppressed metastatic growth of pancreatic carcinoma.	(126)
miR-126-3p	Human BMSCs	Lentivirus	Inhibited proliferation, invasion, and metastasis of cancer cells, and promoted their apoptosis both *in vitro* and *in vivo*	(130)
TRAIL	Human Pancreas	Plasmid	Inhibited viability of pancreatic cancer cells	(153)
TRAIL	Human BMSCs	Plasmid	Increased transfection efficiency and tumor eradication in a xenograft mouse model	(127)
NK4	Rat BMSCs	Adenovirus	Inhibited proliferation and migration of the pancreatic cancer cell line	(136)
miR-1231	Human BMSCs	Direct oligonucleotides	*In vitro*: inhibited the proliferation, migration, invasion, and adhesion to the matrix of PC cells *In vivo*: inhibited tumor growth.	(129)
HSV-TK	Mice BMSCs	Plasmid	Inhibited primary tumor growth and increased survival in tumor models.	(132)
IFN- β	Human BMSCs	Adenovirus	Suppressed tumor growth *in vitro* and *in vivo*	(134)
HSV-TK	Mice BMSCs	Plasmid	Reduced tumor growth and liver metastases.	(133)
TRAIL	Human ATMSCs	Retrovirus	Increased apoptosis in cell line and tumor eradication in a mouse model, mediated apoptosis without significant toxicity to normal tissues.	(80)
NIS	Mice BMSCs	Plasmid	Significant delay and reduction in tumor growth	(154)
TRAIL	Human ATMSCs	Lentivirus	Inhibited tumor growth and reduced tumor size	(128)
IL15	Human UCMSCs	Lentivirus	Signiﬁcantly inhibited tumor growth and prolonged the survival of tumor-bearing mice	(135)
TRAIL	Human BMSCs	Adenovirus	Eradicated TRAIL-resistant pancreatic carcinoma cells in combination with XIAP inhibitor.	(81)

BMSCs: Bone marrow-derived MSCs; UCMSCs: Umbilical cord-derived MSCs; ATMSCs: Adipose tissue-derived MSCs; MSCs: Mesenchymal stem cells

**Table 5:** Application of genetically modified MSCs in other GI cancer therapy

**Cancer**	**Transfected gene**	**Source of MSCs**	**Vector**	**Anti-cancer effects**	**ref**
OC	TRAIL	Human BMSCs	Lentivirus	Increased cancer cell apoptosis *in vitro* and significantly reduce tumor growth and metastasis *in vivo*.	(137)
OC	IFN-β	Human GMSCs	Lentivirus	Inhibited proliferation of TSCC cells and increased apoptosis. Decreased tumor volume and lowered number of cancer cells *in vivo*.	(139)
OC	TRAIL	Human GMSCs	Lentivirus	*In vitro*: increased cell death and apoptosis. *In vivo*: reduced and inhibited TSCC growth.	(138)
ESCC	miR-375	Human UCMSCs	Plasmid	Suppressed ESCC cell proliferation, invasion, migration, tumorsphere formation, and increased apoptosis *in vitro,* furthermore decreased tumor growth *in vivo*.	(141)
ESCC	TRAIL	Human BMSCs	Adenovirus	Inhibited proliferation and induced apoptosis *in vitro* also repressed tumor growth *in vivo*	(140)
GC	CD	Human BMSCs	Plasmid	Anticancer activity in cell line and decreased tumor volume in a mouse model	(142)
GC	TNF-α	Human UCMSCs	Lentivirus	Inhibited gastric cancers growth *in vivo*	(143)
GC	LIGHT (TNFSF14)	Human UCMSCs	Lentivirus	Inhibited tumor growth and increased necrosis *in vivo*.	(146)
GC	NK4	Human BMSCs	Lentivirus	Migration ability and anti-cancer activity in cell line and gastric cancer xenografts model.	(147)

OC: Oral cancer; ESCC: Esophageal squamous cell carcinoma; GC: Gastric cancer; BMSCs: Bone marrow-derived MSCs; UCMSCs: Umbilical cord-derived MSCs; GMSCs: Gingival-derived MSCs; MSCs: Mesenchymal stem cells; GI cancer: Gastrointestinal cancers
